# Decoding the Invisible: A Diagnostic Odyssey of Pulmonary Alveolar Microlithiasis From the Desert State of India, Rajasthan

**DOI:** 10.7759/cureus.101414

**Published:** 2026-01-13

**Authors:** Ramakant Dixit, Mukesh Goyal, Deepak Suthwal, Komal Srivastav, Ranjeet Meghwanshi

**Affiliations:** 1 Department of Respiratory Medicine, Jawaharlal Nehru Medical College, Ajmer, IND

**Keywords:** black pleura sign, depositional lung disorders, intra-alveolar calcification, pulmonary alveolar microlithiasis, transbronchial lung biopsy

## Abstract

Pulmonary alveolar microlithiasis (PAM) is a rare depositional disorder that carries an autosomal recessive pattern of inheritance. It may present at any age and is usually detected incidentally with an abnormal chest X-ray. Most patients are asymptomatic or have minimal symptoms at the time of detection of the disease. Here, we report a case of a 30-year-old female patient who presented with an illness duration of six months and complaints of dry cough, chest pain, and shortness of breath. She was started on anti-tubercular treatment on clinical grounds by her physician, but was not relieved of her symptoms. This case highlights the importance of having PAM in the differential diagnosis. This is, to the best of our knowledge, the first case of PAM from Rajasthan, a desert state of India, in spite of many institutional and monetary challenges.

## Introduction

Pulmonary alveolar microlithiasis (PAM) is a rare deposition disorder with an autosomal recessive pattern. It is characterized by intra-alveolar accumulation of calcified concretions (referred to as microliths) in the absence of any known calcium metabolism disorder [1]. PAM is usually found incidentally with an abnormal chest radiograph in an asymptomatic patient. Presentation may occur at any age, although symptoms usually occur in the third or fourth decade of life [2].

Marco Malpighi, an Italian scientist, was the first to give a concise and detailed macroscopic description in 1686: “*In vesciculis pulmonum innumeri lapilli sunt*” (In the vesicles of the lungs are countless small stones) [3]. In 1918, Norwegian pathologist Francis Harbitz provided an accurate autoptic and radiological description of a second case [4]. The third case was reported in 1932 in Germany by Schildknecht [5], and in 1933, the disease was named PAM by Hungarian pathologist Puhr [6].

PAM is a rare disease with only a few more than 1000 cases reported worldwide. According to available literature, most cases are from Asia (56.3%), followed by Europe (27.8%). However, the exact incidence and epidemiology of PAM in the Indian sub-continent are not known due to the rarity of reports in the literature [7]. No case of PAM has been reported from the western, desert state of Rajasthan, in India, to date. There may be a possible regional variation in the occurrence of this disease, and hence each case of PAM must be reported, as we are still in the infancy of the epidemiology of PAM.

We report here a rare case of academic importance, which, to the best of our knowledge, is the first case from this region. The patient presented with a moderate degree of complaints, was getting adequately treated for a more common disease (tuberculosis), but was not improving. Radiological imaging and histology showed the real picture, leading to the diagnosis.

## Case presentation

A 30-year-old female patient, a homemaker by occupation, presented with chief complaints of dry cough for six months, chest pain for three months, and shortness of breath for two months. On detailed history, she reported being asymptomatic six months back. She then developed a cough that was insidious in onset, gradually progressive, during the initial days more in the early morning and later, on and off throughout the day without any postural or seasonal variation. The chest pain was also insidious in onset, gradually progressive, diffuse in nature, without diurnal/postural variation, but increased on exertion with increased intensity during the winter season. The shortness of breath was insidious in onset, gradually progressive, aggravated on doing household work, while walking, climbing stairs, and relieved on taking rest, without any diurnal or seasonal variation. She was treated for pulmonary tuberculosis with adequate doses for the previous 30 days, on a radiological basis, by her local physician. There was no history of any other prolonged medication in the past. She denied any history of fever, rashes, night sweats, hemoptysis, significant weight loss, and altered bowel/bladder habits. She denied any form of addiction. She had undergone an appendicectomy at the age of 15 years.

**Figure 1:**
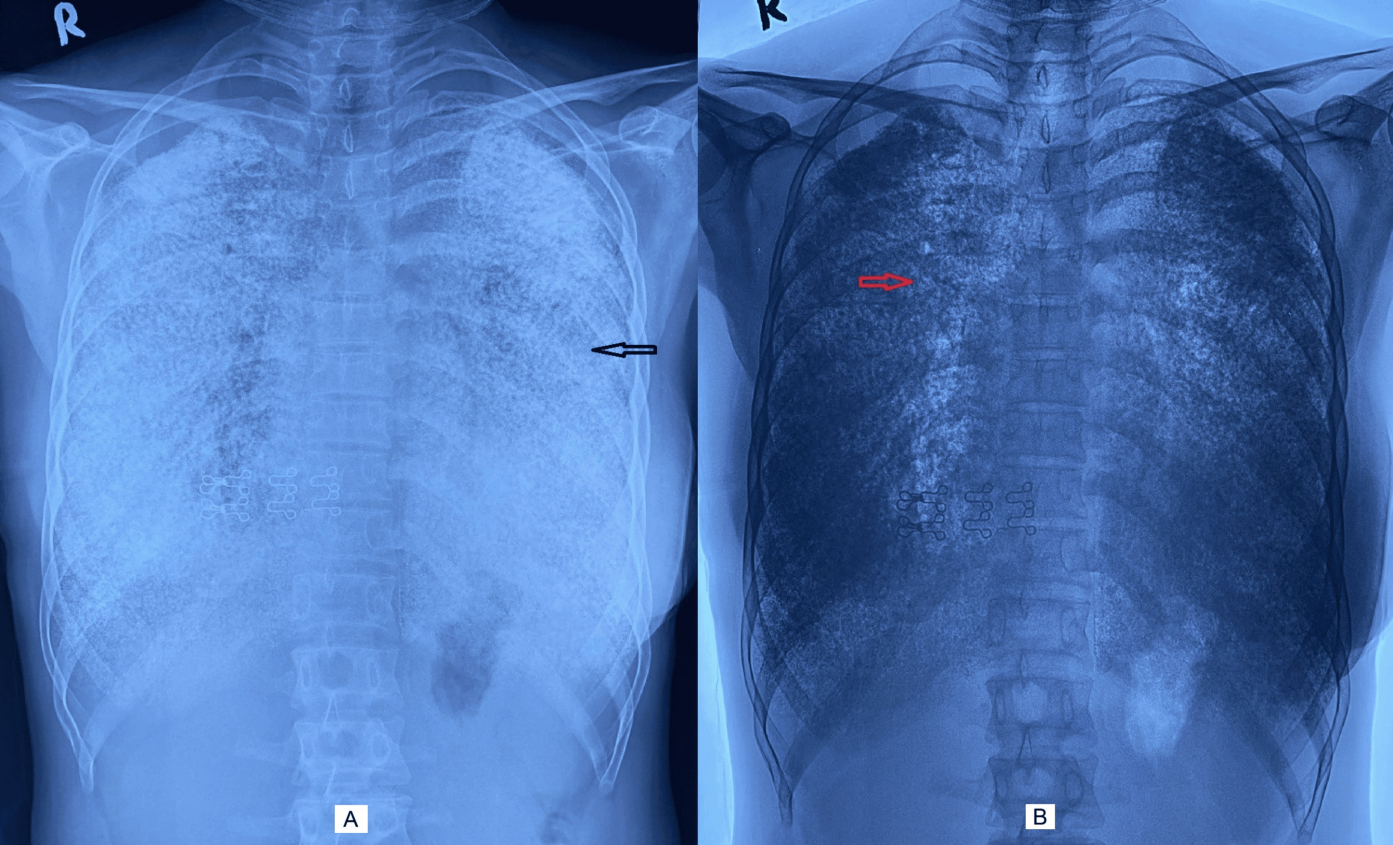
Chest X-ray showing bilateral diffuse sand-like shadow (black arrow, A) with negative image showing pulmonary calcification (red arrow, B)

At presentation at our hospital, general physical and other systemic examinations were essentially normal. A chest X-ray was done, which showed a near complete white-out appearance (Figure 1A), with bilateral fine, high-density small nodular opacities resembling sand-like calcification (Figure 1B) throughout both the lung fields. Routine investigations of blood for hematological and biochemistry workup were within normal range.

**Figure 2:**
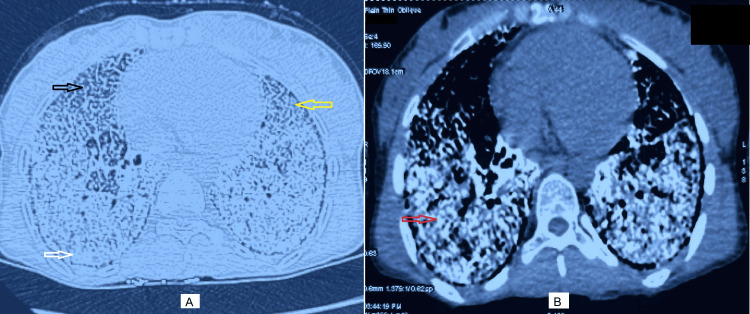
CT scan showing (A) consolidation (white arrow), focal crazy paving (black arrow) and ground glass opacity (yellow arrow), and (B) calcified nodules (red arrow)

High-resolution computed tomography (HRCT) of the chest revealed multiple calcific foci throughout the lungs along with crazy-paving pattern and patchy areas of ground glass opacities with consolidation in bilateral lung fields (Figure 2). The findings raised the suspicion of occupational lung disease or depositional disorder.

Clinical evaluation was not very helpful in making a diagnosis, and occupational history was lacking to point towards any occupational hazard. But keeping in mind that she was receiving anti-tubercular treatment, differentials like tuberculosis, varicella, and parasitic infections were also kept in consideration despite the absence of a history of fever/anorexia, and a fiberoptic bronchoscopy (FOB) was planned. The patient underwent FOB that showed bilateral mobile vocal cords with a normal tracheobronchial tree without any visible endobronchial lesion/external compression. Bronchoalveolar lavage (BAL) and trans-bronchial lung biopsy were performed and subjected to bacterial culture, cartridge-based nucleic acid amplification test (CBNAAT) for tubercular bacilli, cytopathological examination, and for the presence of any parasitic ova/cyst. BAL, CBNAAT, and culture were negative for mycobacteria and other pathogenic organisms. BAL cytology showed predominantly alveolar macrophages, polymorphs with few epithelial cells against a pink amorphous background.

**Figure 3:**
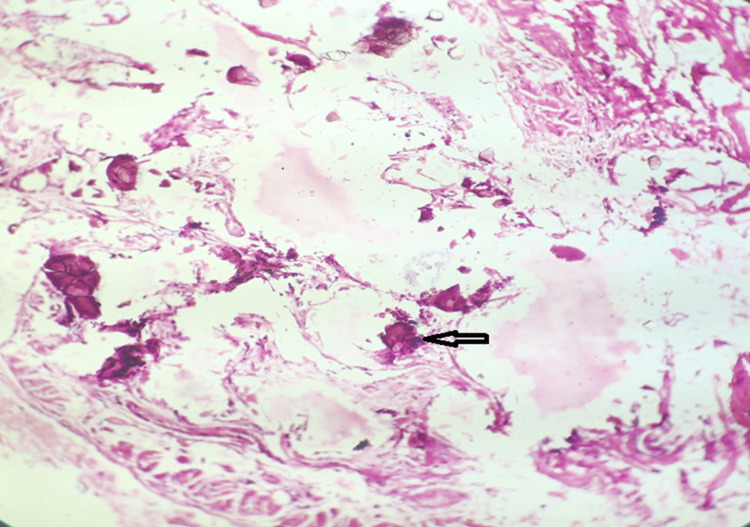
Histopathology showing intra alveolar concentric, lamellated calcium spherules with preserved alveolar architecture (H&E x 10)

The histopathology report from the lung biopsy showed concentric, lamellated calcium spherules within alveolar spaces (Figure 3). The final diagnosis of PAM was made with appropriate clinical, imaging, and histological features. The gene analysis test for PAM could not be done due to financial and institutional constraints.

The patient received symptomatic treatment with bronchodilators and supplemental oxygen, etc. Her symptoms improved during hospitalization. The patient is currently under regular follow-up and clinically stable.

## Discussion

PAM is an autosomal recessive disorder that occurs due to a mutation in *the SLC34A2* (solute carrier family 34 member 2) gene at chromosome 4p15.2 that encodes a sodium-dependent phosphate type 2b (NPT2b) transporter [8]. NPT2b transporter clears phosphate from alveolar spaces into type II pulmonary alveolar cells in the presence of sodium ions at a ratio of 3Na + 1:1HPO4-2 [6]. If this protein is dysfunctional, then homeostasis of phosphate is affected because type 2 alveolar cells are unable to clear phosphate. Impaired phosphate clearance leads to the accumulation of phosphate in the alveoli. Over time, an excess of phosphate forms precipitates with calcium called microliths. These microliths accumulate in the alveoli, causing characteristic radiological findings and eventually impairing lung function.

In this disorder, there is no abnormality of calcium metabolism, despite phosphate elimination being defective, which leads to excess accumulation of phosphate. SLC34A2 is also expressed in other organs, but microliths are usually not formed due to the presence of redundant ion transporters, accounting for the low incidence of extrapulmonary manifestations in PAM. NPT2b is most abundantly expressed in the lung and small intestine, but the gene is also expressed in the thyroid, salivary gland, mammary gland, uterus, and testes [9,10].

PAM has an indolent course in nature. Despite dramatic imaging findings on chest radiograph, most of the patients are asymptomatic at the time of diagnosis, reflecting clinical-radiological dissociation. PAM is often found incidentally on imaging studies performed for other reasons. Symptoms usually develop during the third or fourth decade of life, although presentation at extreme age is also possible. In these situations, presentation is more acute and aggressive, which can lead to acute respiratory failure. Common symptoms include dyspnea, non-productive cough, chest pain, and asthenia. Less frequent symptoms are sputum production, hemoptysis, and fatigue. Usually, laboratory investigations are normal and not very helpful in making the diagnosis [11].

An abnormal X-ray is the first clue to suspect PAM, especially in asymptomatic patients. On X-ray, the classical findings are small, diffuse, scattered innumerable micronodules with predilection towards bases compared to apices and typically called “sandstorm appearance”, as seen in our case also. PAM is also observed in children (youngest patient diagnosed at eight months of age [12]); however, ground glass opacities are not infrequent as compared to calcification and are usually restricted to lower zones [13]. As the disease progresses, the density of the lung parenchyma may obscure the cardiac borders, diaphragms, costophrenic sinuses, and cardio-phrenic sulci, resulting in the radiographic manifestation described as the “vanishing heart phenomenon" [7]. This phenomenon is non-specific and can be seen in any cause of acute respiratory distress syndrome.

HRCT is the most useful diagnostic imaging modality available. It shows bilaterally symmetric involvement with predominant mid and lower lung involvement. Diffuse calcified pulmonary nodules, subpleural cyst, diffuse ground glass opacity, inter-septal septal thickening, consolidation, crazy-paving pattern, and emphysema are commonly seen on HRCT scan in such cases. Pulmonary calcified nodules are most prominent in the peripheral, mediastinal, and fissure subpleural regions, and each lobe appears surrounded by a fine, dense outline. Castellan et al. [14] called it “stony lung,” while Bendstrup [1] called it “white-out lung” to describe this condition. Ground glass opacities and small parenchymal nodules were present as the predominant tomographic findings in 100% of cases in one study [2]. “Black pleura” sign, which is subpleural cysts separated from the underlying calcified lung parenchyma, is classically described in this condition. Interlobular septal thickening producing “crazy-paving pattern” may also be seen in PAM, although on mediastinal windows, the calcifications are seen tracing septal lines, which can help distinguish this process from pulmonary alveolar proteinosis [15]. In cases where imaging features are inconclusive, lung biopsies need to be performed. Histopathological examination of lung biopsy samples confirms the presence of microliths.

No effective medical or gene therapy currently exists for PAM. Palliative treatments for this condition include systemic corticosteroids, calcium-chelating agents, and serial bronchopulmonary lavage, but do not show any benefit. Lung transplantation remains the only definitive treatment for end-stage PAM.

PAM generally has a slow but progressive course. Pulmonary function tests often remain normal till disease becomes advanced. Advanced disease could result in severe respiratory insufficiency and right heart failure. Despite slow progression, prognosis in advanced cases remains poor, with significant morbidity and mortality due to respiratory failure. There is no curative treatment for PAM and management mainly focuses on symptom relief and supportive care [11].

## Conclusions

PAM is a rare disease with a global distribution and characteristic radiological features. The diagnosis is suggested by the clinico-radiological dissociation with characteristic radiological features and may be confirmed by lung biopsy. This case highlights the importance of history and clinical examination and also shows that we may need to think out of the box to clinch the diagnosis. Despite its typically slow progression, PAM can lead to severe respiratory impairment in advanced stages. There are still no effective medical therapies, hence management remains supportive. Lung transplantation offers hope for patients with end-stage disease. Advances in genetic testing and imaging techniques have improved diagnostic accuracy. Future research into the underlying pathophysiological mechanisms and the effect of regional or geographical variation, if any, may offer new insights and potential therapeutic targets to improve outcomes for individuals affected by this rare disease.
